# Complement deficiencies and susceptibility to systemic lupus erythematosus revisited

**DOI:** 10.1186/ar3974

**Published:** 2012-09-27

**Authors:** KB Elkon, DM Santer, A Wiedeman

**Affiliations:** 1 University of Washington, Seattle, WA, USA; 2 University of Alberta, Edmonton, AB, Canada

## 

Recent demonstration of the contribution of more than 30 different SNPs to lupus susceptibility has informed our understanding of pathogenesis of disease. Yet each of these genetic variants is commonly found in the general population and contributes a small effect to lupus susceptibility. In contrast, the almost universal association between C1q deficiency and SLE, as well as high relative risk of other classical components, provides a special opportunity to understand mechanisms of disease.

The complement pathway was implicated in the immunopathogenesis of lupus and other autoimmune disorders decades ago. The apparent paradox that early complement component (C1q, C2 and C4) deficiencies predispose to lupus has been explained by the beneficial roles of these proteins in promoting the clearance of apoptotic cells and immune complexes (ICs). We recently showed that, in the absence of C1q, instead of ICs binding to monocytes, they preferentially engage plasmacytoid dendritic cells (pDC) so providing a powerful stimulus for the production of IFNα, the cytokine with potent immune adjuvant properties [1,2]. We confirmed and extended these findings using microarray analysis of total peripheral blood mononuclear cells and purified monocytes following incubation with SLE ICs in the presence or absence of C1q [3]. We observed that C1q suppressed SLE IC-induced interferon-stimulated genes such as TNFSF13B (BAFF) and TNFSF10 (TRAIL), which are associated with SLE pathogenesis. Interferon-independent pathways that were differentially affected by the presence or absence of C1q in SLE ICs included: multiple cytokines/chemokines (for example, CCL20, CCL23), receptors (for example, CD36, STAB1), and enzymes (for example, RNASE1,2,6, SOD2). Exposure of monocytes to SLE ICs was surprisingly non-inflammatory even when gene expression was examined by microarray.

How then are the lower frequencies of lupus in C4, C2 and C3 deficient patients explained? First, isolated C1q has been shown to exert immunosuppressive properties that have not been identified with C4 or C2. Also, we and others have suggested that C3b may be the key complement protein required for removal of apoptotic cells but that C4b could potentially function in this regard. The lower prevalence of SLE in individuals with a deficiency of C4, C2 or C3 could then be due to only one pathway being defective and, possibly, a functional role for C4b in protection. Future studies will continue to address these questions and guide interventions to promote the safe handling of apoptotic debris and ICs.
